# An Overview of the Ultrawide Bandgap Ga_2_O_3_ Semiconductor-Based Schottky Barrier Diode for Power Electronics Application

**DOI:** 10.1186/s11671-018-2712-1

**Published:** 2018-09-19

**Authors:** HuiWen Xue, QiMing He, GuangZhong Jian, ShiBing Long, Tao Pang, Ming Liu

**Affiliations:** 1 College of Mechanical and Electrical Engineering, Sichuan Agricultural University, Yaan, 625014 China; 2 Key Laboratory of Microelectronic Devices & Integration Technology, Institute of Microelectronics of Chinese Academy of Sciences, Beijing, 100029 China; 3 School of Microelectronics, University of Science and Technology of China, Hefei, 230026 China

**Keywords:** Gallium oxide (Ga_2_O_3_), Ultrawide bandgap semiconductor, Power device, Schottky barrier diode (SBD), Breakdown electric field, Baliga’s figure of merit, On-resistance

## Abstract

Gallium oxide (Ga_2_O_3_) is a new semiconductor material which has the advantage of ultrawide bandgap, high breakdown electric field, and large Baliga’s figure of merit (BFOM), so it is a promising candidate for the next-generation high-power devices including Schottky barrier diode (SBD). In this paper, the basic physical properties of Ga_2_O_3_ semiconductor have been analyzed. And the recent investigations on the Ga_2_O_3_-based SBD have been reviewed. Meanwhile, various methods for improving the performances including breakdown voltage and on-resistance have been summarized and compared. Finally, the prospect of Ga_2_O_3_-based SBD for power electronics application has been analyzed.

## Background

With the fast development of electrical power, industrial control, automotive electronics, and consumer electronics industries, there is a huge demand for high-performance power semiconductor devices. Wide and ultrawide bandgap semiconductor materials can satisfy this demand [1, 2]. Among the five structures of Ga_2_O_3_ single crystal, monoclinic *β*-Ga_2_O_3_ is the most stable, and it has an ultrawide bandgap (*E*_g_~ 4.8 eV) and very high breakdown electric field (*E*_br_~ 8 MV cm^−1^), compared to the traditional Si and later developed SiC and GaN material. In consequence, *β*-Ga_2_O_3_ shows a much large Baliga’s figure of merit (BFOM =$\varepsilon \mu {E}_{\mathrm{b}}^3$; *ε* is the relative dielectric constant, and *μ* is the electron mobility). BFOM is an important criterion to assess the appropriateness of a material for power device application [3–11]. Table 1 compares the basic physical properties of Si, wide bandgap (GaN, SiC), and ultrawide bandgap (*β*-Ga_2_O_3_) semiconductor material. Furthermore, for the growth of single-crystal *β*-Ga_2_O_3_ substrate, there are easy, low-cost, and mass-producible melt-growth methods at atmospheric pressure, such as floating zone (FZ) [12, 13] and the edge-defined film-fed growth (EFG) [14–17]. This is another superiority of Ga_2_O_3_ in the aspect of high-quality single-crystal growth, compared with SiC and GaN. Therefore, *β*-Ga_2_O_3_ is a promising candidate for next-generation high-power semiconductor devices such as Schottky barrier diode (SBD) [18–24] and metal-oxide-semiconductor field-effect transistor (MOSFET) [25–29]. It is worth noting that a lot of studies on the Ga_2_O_3_ material growth and power device fabrication and characterization have been carried out in the last several years, so in this paper, we reviewed the material properties of the ultrawide bandgap Ga_2_O_3_ semiconductor and the investigations of the Ga_2_O_3_-based SBD for power electronics application. In SBD, the most important performance parameters are breakdown voltage (*V*_br_) and on-resistance (*R*_on_), so through summarizing and comparing the various methods for improving the *V*_br_ and *R*_on_ performances, we wish our reviewing work is beneficial for the development of Ga_2_O_3_-based power devices.

**Table 1:** Comparison of the physical properties of Si, GaN, SiC, and *β*-Ga_2_O_3_ semiconductor [5]

Semiconductor material	Si	GaN	4H-SiC	*β*-Ga_2_O_3_
Bandgap *E*_g_ (eV)	1.1	3.4	3.3	4.7–4.9
Electron mobility *μ* (cm^2^ V^−1^ s^−1^)	1400	1200	1000	300
Breakdown electric field *E*_br_ (MV/cm)	0.3	3.3	2.5	8
Baliga’s FOM ($\varepsilon \mu {E}_{\mathrm{b}}^3$)	1	870	340	3444
Thermal conductivity *λ* (W cm^−1^ K^−1^)	1.5	2.1	2.7	0.11

## Physical Properties of Gallium Oxide Semiconductor

Gallium oxide (Ga_2_O_3_) is a new oxide semiconductor material, but it has a long research history. The investigation on the phase equilibria in the Al_2_O_3_-Ga_2_O_3_-H_2_O system can be traced back to 1952, and R. Roy et al. determined the existence of polymorphs of Ga_2_O_3_ and their stability relations [30]. In 1965, H. H. Tippins et al. studied the optical absorption and photoconductivity in the band edge of *β*-Ga_2_O_3_ and confirmed its bandgap with a value of 4.7 eV [6]. In 1990s, a number of methods on the melting growth of Ga_2_O_3_ bulk single-crystal and epitaxial growth of Ga_2_O_3_ film had been developed. In recent 5 years, owing to its special properties and the successful growth of high-quality and large-size single-crystal substrate, Ga_2_O_3_ material has attracted a lot of research interest.

**Fig. 1:**
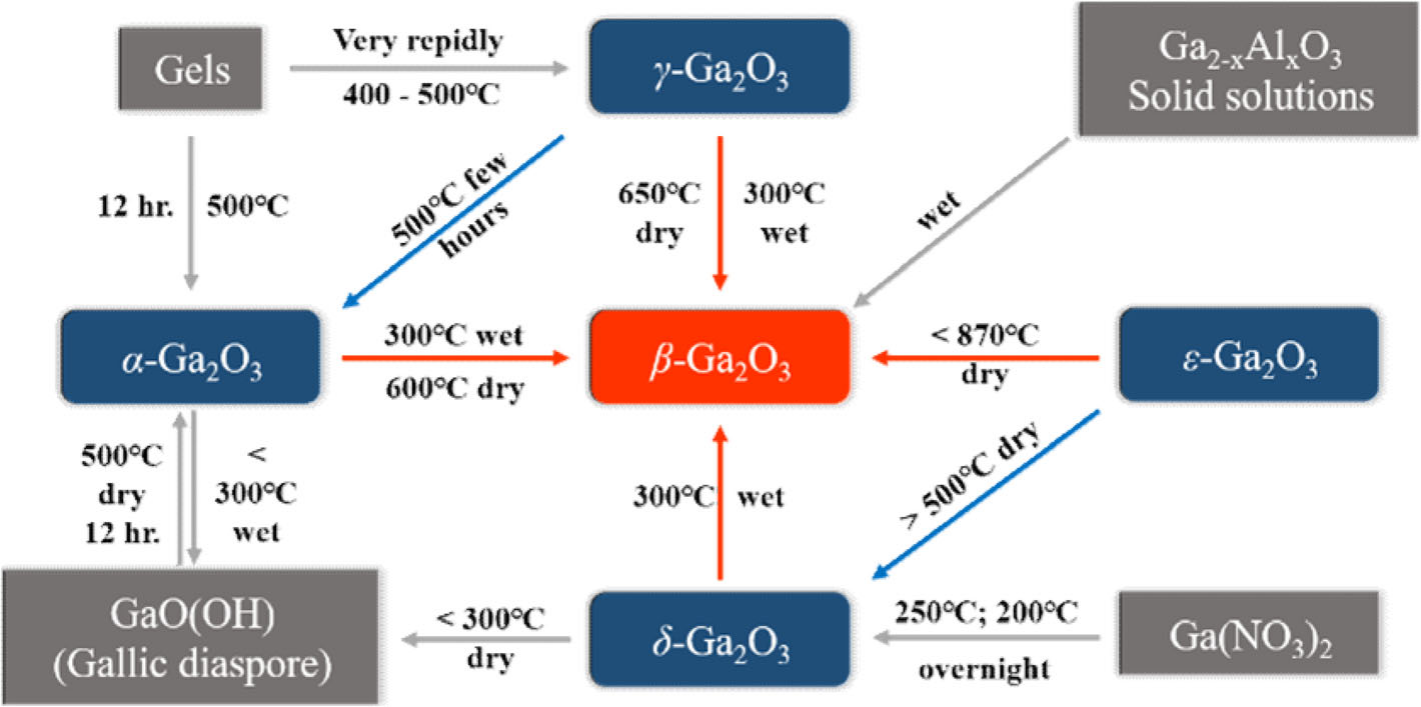
Transformation relationships among the crystalline phases of Ga2O3 and their hydrates [30]

Till now, people have found five crystalline phases of Ga_2_O_3_, i.e., *α*, *β*, *γ*, *δ*, and *ε* phases. The transformation relationships among these five phases are shown in Fig. 1 [30]. The monoclinic phase *β-*Ga_2_O_3_ has the best thermal stability, while the other four phases are metastable and are apt to transform to *β-*Ga_2_O_3_ at high temperatures. Therefore, at present, most studies focus on *β-*Ga_2_O_3_. Some recent investigations also found that other phases presented some special material properties which *β* phase did not have. For example, *α-*Ga_2_O_3_ has a corundum crystal structure similar to that of sapphire (Al_2_O_3_), so it is comparatively easy to epitaxially grow high-quality *α-*Ga_2_O_3_ single-crystal film on the currently existing Al_2_O_3_ single-crystal substrate. Hexagonal phase *ε-*Ga_2_O_3_ is the second stable phase and presents strong spontaneous polarization effect which is beneficial to form high-density 2D electron gas in the heterojunction interface [31], similar to the condition in AlGaN/GaN junction. In recent years, due to the successful growth of large-size *β-*Ga_2_O_3_ single-crystal substrate and its best stability, up to now, the studies on *β-*Ga_2_O_3_ are far more than those on the other four phases. So, in this paper, we mainly review the research works on *β-*Ga_2_O_3_.

**Fig. 2:**
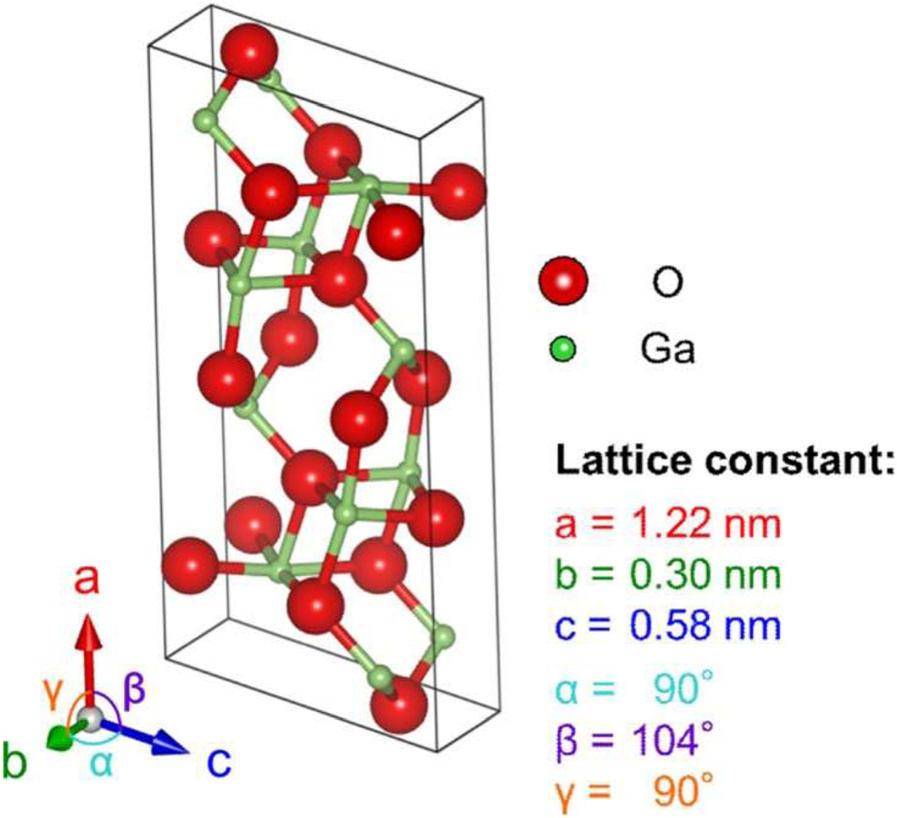
The lattice structure of β-Ga_2_O_3_ crystal. Reprinted from ref. [5]

*β-*Ga_2_O_3_ belongs to monoclinic system and is thermally stable. Its lattice constants are *a* = 1.22 nm, *b* = 0.30 nm, and *c* = 0.58 nm, as shown in Fig. 2. The crystalline structure of *β-*Ga_2_O_3_ determines that it has a certain conductivity, but which is limited by its ultrawide bandgap (4.7–4.9 eV), the widest one of all the known transparent semiconductor materials. Only if some defect energy levels exist in the bandgap and free electrons generate, the material has comparatively strong conductivity. For most wide bandgap semiconductors, conductivity is formed just because of the existence of defect levels in bandgap, such as ZnO semiconductor [32]. The intrinsic electrical conduction of *β-*Ga_2_O_3_ originates from the free electrons led by the point defects formed in the bulk of crystal. Most studies have demonstrated that the oxygen vacancies are the key defects for the electrical conduction [33–35].

It is interesting that due to the existence of the plenty of oxygen vacancies in polycrystalline *β-*Ga_2_O_3_, it is easy to absorb some kind of gas to make the resistivity change, so there have been many reports about using *β-*Ga_2_O_3_ to fabricate gas sensors for the detection of H_2_, CH_4_, CO, and O_2_ [36–39]. In addition, because the lattice constant of *β-*Ga_2_O_3_ in [100] direction is much larger than those in [001] and [010] directions, it is easy to peel off ultrathin film along [100] direction for device fabrication [27, 40–43]. At the same time, owing to this crystal structure characteristic, in the fabrication of *β-*Ga_2_O_3_ wafers, cutting the bulk along [100] direction can acquire flat surface with very low roughness.

**Fig. 3:**
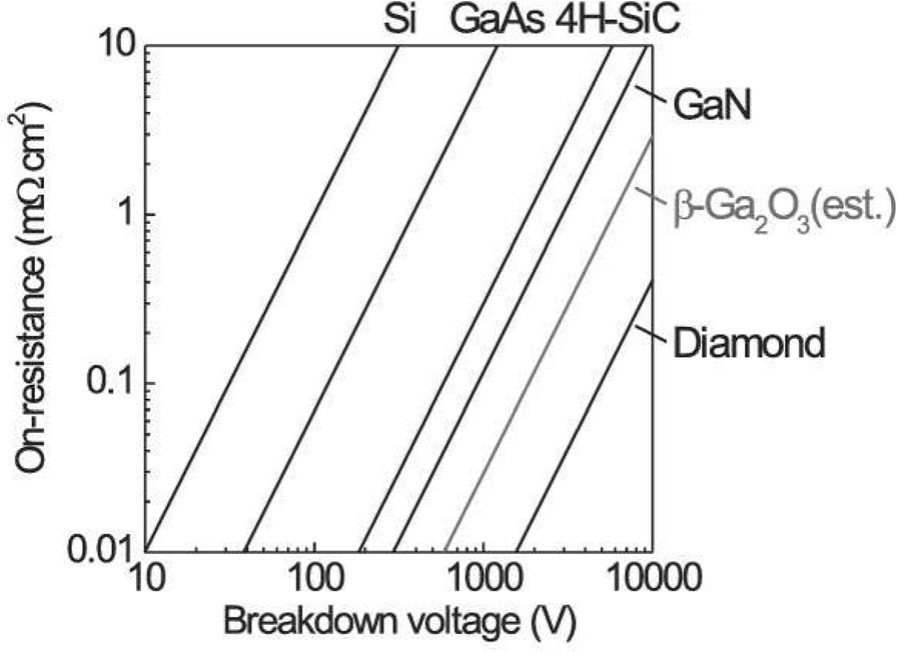
Theoretical limits of on-resistance (*R*_on_) as a function of breakdown voltage (*V*_br_) for β-Ga_2_O_3_ and representative semiconductors. Reprinted from ref. [26]

Compared to SiC and GaN, *β-*Ga_2_O_3_ possesses particular electrical characteristics, among which the ultrawide bandgap (4.7–4.9 eV) is the most prominent. This makes it have a very high critical breakdown electrical field (*E*_br_≈8 MV/cm), about twice those of SiC and GaN. The breakdown electrical field of material is a very important parameter for unipolar power devices. If a material has higher *E*_br_, in the material of unit thickness, higher electrical field can be maintained, which is advantageous for the reduction of device size and enhancement of the integration level of power modules. Figure 3 shows the fundamental limits of on-resistance (*R*_on_) as a function of breakdown voltage (*V*_br_) for several important semiconductors including Si, GaAs, SiC, GaN, Ga_2_O_3_, and diamond [5]. From this figure, we can find that the conduction loss of Ga_2_O_3_ devices is one order of magnitude lower than those of SiC and GaN devices at the same *V*_br_. Thus, Ga_2_O_3_ shows its great potential in unipolar devices. As the saturation electron mobility of *β-*Ga_2_O_3_ is comparatively low (~ 200 cm^2^ V^*−*1^ s^*−*1^), *β-*Ga_2_O_3_ is not suitable for high-frequency devices compared to GaN. However, its wide bandgap can compensate for this disadvantage since thinner drift layer has smaller depletion width; thus, the parasitic capacitance can be decreased to meet the requirements of high-frequency applications. Besides, the bandgap of about 4.8 eV makes Ga_2_O_3_ possess special absorption wave band (250–280 nm) which is just located in the range of solar blind ultraviolet (UV) ray, so Ga_2_O_3_ is a natural good material for fabricating UV detectors [44–47].

In recent years, the n-type doping of *β-*Ga_2_O_3_ has been basically realized. Si and Sn elements, as its donor impurities with shallow energy level, have low-activation energy. Doping concentration can be well modulated to be in the range of 10^15^–10^19^ cm^−3^ [47], with the highest value of 10^20^ cm^−3^ reached. At the same time, with the change of doping concentration, the optical and electrical properties will also change. For example, the resistivity of n-type *β-*Ga_2_O_3_ changes in the range of 10^−3^ − 10^12^ Ω cm with the changing doping concentration [48, 49]. The bandgap also changes with different doping concentration, so the light absorption characteristics of Ga_2_O_3_ are influenced [50].

From the development of Ga_2_O_3_, this material still has some disadvantages as follows. (1) P-type doping is a big challenge of Ga_2_O_3_ material. Because the acceptor levels are far from the valence band of *β-*Ga_2_O_3_, the activation energy of acceptor impurities is very high. Meanwhile, the n-type background impurities in Ga_2_O_3_ crystal will also produce self-compensation effect on acceptor impurities, resulting to the self-insulating of the material. Therefore, there still has been no effective p-type doing. (2) The thermal conductivity of Ga_2_O_3_ is too low. Experimental and theoretical investigations have proved that the thermal conductivity of *β-*Ga_2_O_3_ is just only 0.1–0.3 W cm^−1^ K^−1^ [51–53]. This is adverse to the power device used in high-voltage and high-current circumstance. Excessive heat accumulation will seriously affect the performance and reliability of the device. (3) Carrier mobility is low. The theoretical mobility of Ga_2_O_3_ is limited to about 200 cm^2^/V s due to the influence of scattering [54]. Low mobility has a negative impact on the frequency and current characteristics.

## The Basic Concept of Schottky Barrier Diode

Schottky contact, ohmic contact, and electrical field distribution are the key factors in SBD to attain high performances including on-resistance (*R*_on_) and breakdown voltage (*V*_br_), so various methods for improving them are especially important.

According to the concept of Schottky barrier, the barrier height is related to the work function of Schottky metal and the electron affinity of semiconductor. The work function of different metals changes periodically, and metal needs to have larger work function than semiconductor in order to form Schottky barrier. Nickel (Ni) and platinum (Pt) are the common Schottky metals for *β-*Ga_2_O_3_, and their barrier heights are derived with diverse methods [55–77]. The depletion region under the surface of semiconductor needs necessary thickness to prevent carrier tunneling, and this requires the limited doping concentration of semiconductor. Common values of doping concentration are 10^16^–10^17^ cm^−3^ in the *β-*Ga_2_O_3_ substrate or epitaxy layer [56–62]. The barrier height is actually affected by the interface states and deviates from a simple relationship with work function. The surface pre-treatment aims to reduce the interface states, including the near-surface oxygen vacancies and dangling bonds [78].

The ohmic contact is the basic link between metal and semiconductor. A low-specific resistance of ohmic contact is helpful for the devices to decrease contact resistance (*R*_s_) and on-resistance (*R*_on_). The traditional methods to achieve low-contact resistance are choosing low-work function metal and heavy doping. In fact, the work function of contact metal is always uninfluential for the formation of ohmic contact due to the pinning of interface states. The heavy doping of semiconductor becomes the primary technique for the ohmic contact. The main targets are improving the concentration of carriers and lowering the interface barrier. The RTA (rapid thermal annealing) improves the interfacial characteristics and redounds to reducing the contact resistance. Y. Yao et al. tested nine metals as ohmic contact metals to the *β-*Ga_2_O_3_ and found that titanium (Ti) and indium (In) show good ohmic behavior under specific conditions [79]. After annealing in high temperatures, only titanium can maintain the continuous morphology. Similar to this, most studies applied titanium as the ohmic contact metal with *β-*Ga_2_O_3_ and obtained favorable device performances [60–70].

The breakdown behavior is related to the distribution of electric field inside the devices, and cylindrical junction and spherical junction have larger electric field than parallel-plane junction in the same condition [1]. Therefore, some edge termination protection methods are needed such as field plate to enhance the breakdown voltage [19, 23, 68]. The interface states referred as interface charges normally impact the electric field close to the semiconductor surface and cause the premature breakdown. The leakage current is the indicator of breakdown behavior and is commonly affected by the internal defects of semiconductor, including dislocations. Both situations cause the instability of devices and may decrease the breakdown voltage which should be avoided. The common practice for reducing the impact of interface states is surface passivation, and high-quality substrate is also required for increasing the breakdown voltage.

## Schottky Barrier Diode Based on *β-*Ga_2_O_3_

The difficulties in the growth of high-quality and low-cost single-crystal substrates have affected the commercialization of SiC and GaN devices. While Ga_2_O_3_ single-crystal substrates can be grown by low-cost melting method, the power devices based on Ga_2_O_3_ single crystal have attracted much attention in recent years. At present, the n-type doping technology of Ga_2_O_3_ is quite mature, but the lack of p-type doping makes Ga_2_O_3_ unable to be applied into bipolar devices. The ultra large bandgap makes it a big advantage in the application of unipolar devices. Therefore, the development of the Ga_2_O_3_ power devices is dominated by two kinds of unipolar devices, i.e., the Schottky barrier diode (SBD) and metal-oxide-semiconductor field-effect transistor (MOSFET) [23, 55, 56].

**Fig. 4:**
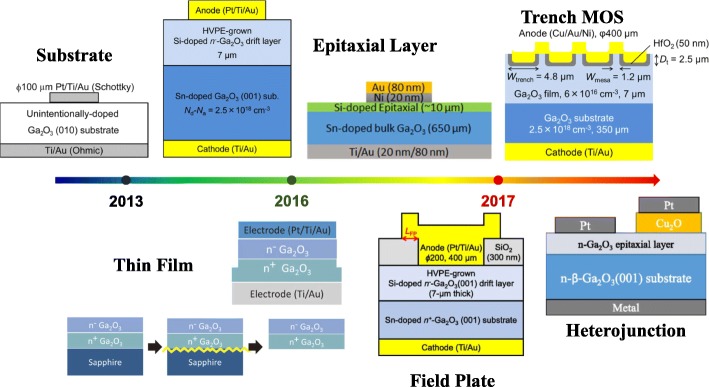
The development of Ga_2_O_3_ SBD in recent years [16, 18, 62, 68–71]

With the development of the wide bandgap (WBG) semiconductor material technology, the SBD device based on WBG semiconductor begins to replace p-n junction diode to apply into power electronic system because no minority carrier storage effect exists in SBD and its switching loss is quite low. In theory, compared to SiC and GaN SBD, Ga_2_O_3_ SBD can achieve the same breakdown voltage with much thinner drift layer. At the same time, thinner drift layer makes lower parasitic capacitance, shortening the reverse recovery time of the device. The main development progress of Ga_2_O_3_ SBD is shown in Fig. 4. With the development of the epitaxy technology, the SBD structure has developed from the initial substrate-based simple structure to the substrate and epitaxial film-based complex structure. Subsequently, through the gradual exploration on the device fabrication processes, advanced terminal structures including filed plate and trench have appeared, further enhancing the device performances. Ga_2_O_3_ SBD starts to present its potential in power electronics applications.

As a new wide bandgap semiconductor material, people confronted a lot of basic problems in the initial development stage of Ga_2_O_3_, so the development progress of Ga_2_O_3_ SBD reflexes the evolution of power SBD very well. The most important part in SBD is the Schottky junction, so in the early research works on Ga_2_O_3_ SBD, there are a substantial numbers of ones focusing on the study on the Schottky junction, mainly including the contact between Ga_2_O_3_ and different Schottky electrodes (Ni、Cu、Au、Pt、TiN) [57–59], the electron transport mechanism of the Schottky junction, the issues of interface states, barrier inhomogeneity and image force existing in the Schottky contact, and the methods of how to acquire perfect ohmic contact in the cathode interface [60, 61].

With the gradual perspicuousness of the physical properties and the increasingly improvement of the fabrication processes, the device performances are progressively enhanced. The following are some typical works in the development of Ga_2_O_3_ SBD.

**Fig. 5:**
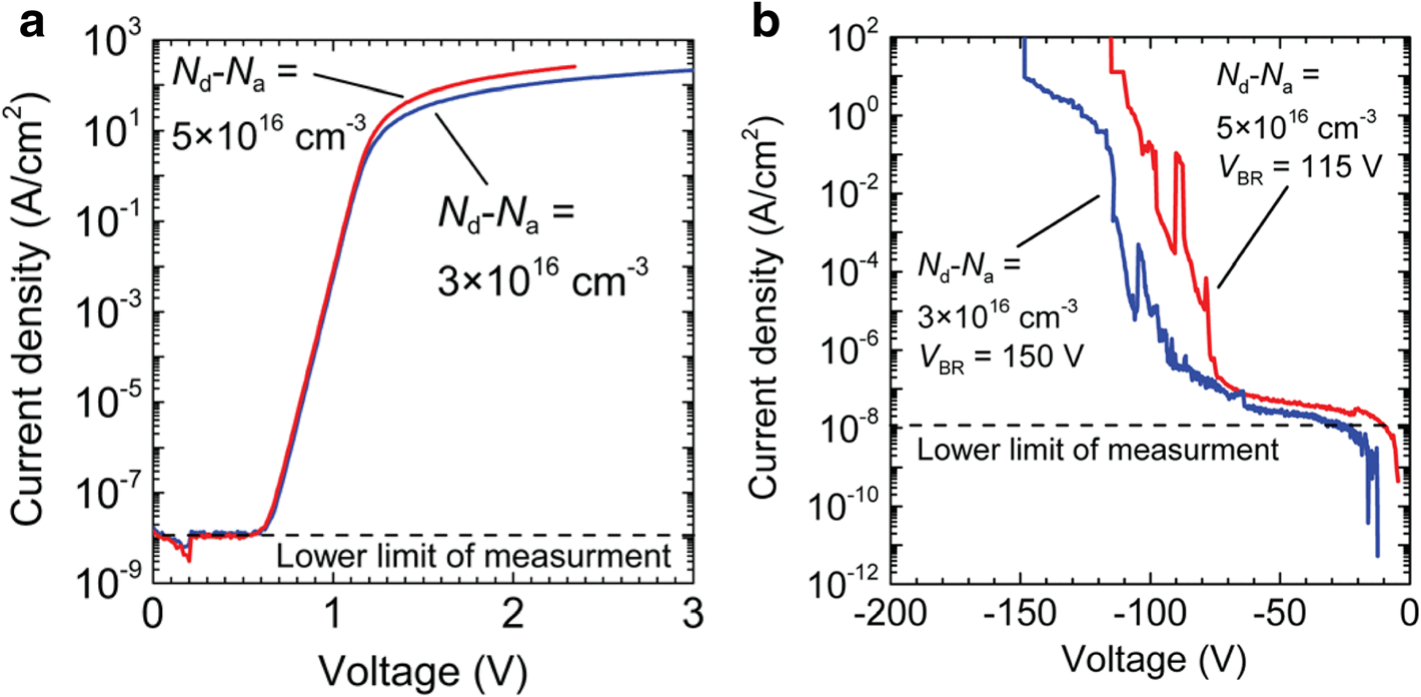
**a**, **b** Forward and reverse electric characteristics of the SBD based on (010) *β*-Ga_2_O_3_ substrates with different doping concentrations. The lower limit of current density measurment is 10^-8^ A/cm^2^. Reprinted from ref. [62]

In 2013, K. Sasaki et al. in Tamura Corporation fabricated SBD based on high-quality (010) *β*–Ga_2_O_3_ single-crystal substrate grown by floating zone method [62]. They investigated the impact of the different doping concentration in the substrate on the device performance and found that higher doping concentration induced lower on-resistance but lower reverse breakdown voltage and larger reverse leakage current. Figure 5 shows the reverse breakdown characteristics of the two SBDs fabricated with (010) *β*–Ga_2_O_3_ substrates with different doping concentrations. The breakdown voltage reaches 150 V. The ideality factor of both devices is close to 1. And the Schottky barrier height of the Pt*/β*–Ga_2_O_3_ interface was estimated to be 1.3–1.5 eV.

**Fig. 6:**
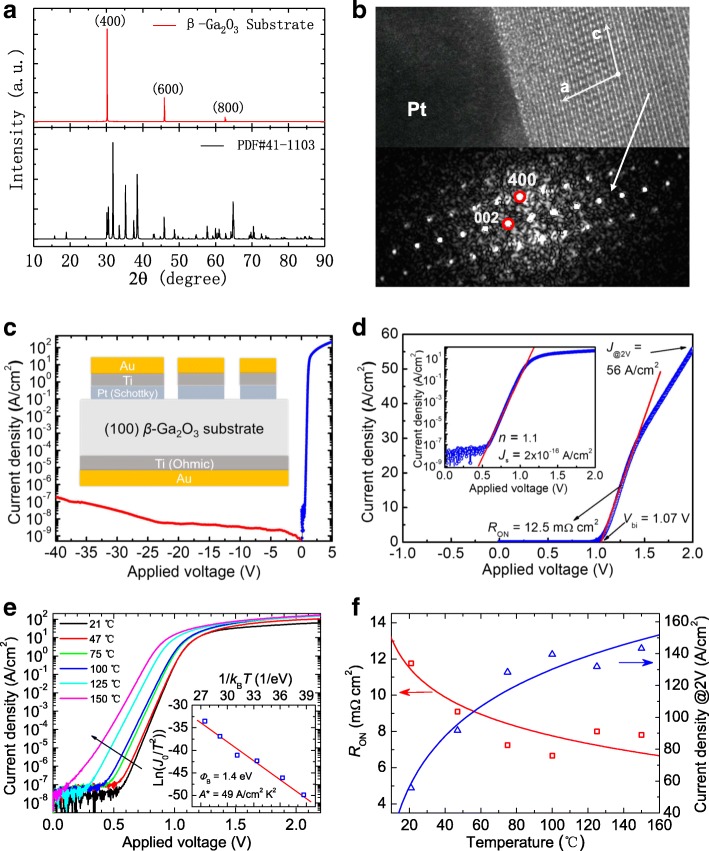
**a** X-ray diffraction (XRD) curve of (100) *β*-Ga_2_O_3_ single-crystal substrate, clearly showing the peaks of (400), (600), and (800) planes. **b** Cross-sectional high-resolution transmission electron microscope (HRTEM) image of Pt/*β*-Ga_2_O_3_ Schottky contact and fast Fourier transformed (FFT) micrograph of *β*-Ga_2_O_3_ crystal. **c** Forward and reverse J–V curve of a Pt/*β*-Ga_2_O_3_ SBD and the schematic of the SBD (inset). **d** Forward J–V curve in linear and semi-logarithmic plot. **e** Temperature-dependent J–V curves and the Richardson’s plot (inset). **f** Dependence of ON-resistance and forward current density on temperature. Reprinted from ref. [60]

Researchers from Institute of Microelectronics of Chinese Academy of Sciences (IMECAS) and Shandong University have collaborated to investigate the SBD based on (100)-oriented *β*–Ga_2_O_3_ bulk substrate. In 2017, they reported a Pt/*β*–Ga_2_O_3_ SBD and its temperature-dependent electrical characteristics. X-ray diffraction (XRD) and high-resolution transmission electron microscopy (HRTEM) analysis showed that the *β*–Ga_2_O_3_ bulk single crystal grown by edge-defined film-fed growth (EFG) technique presented good (100) orientation and good crystal quality (Figs. 6a, b). Through I–V measurements and thermionic emission modeling, the fabricated Pt/*β*–Ga_2_O_3_ SBD device exhibited good performances, including rectification ratio of 10^10^, ideality factor (*n*) of 1.1, Schottky barrier height (*Φ*_B_) of 1.39 eV, threshold voltage (*V*_bi_) of 1.07 V, on-resistance (*R*_on_) of 12.5 mΩ cm^2^, forward current density at 2 V (*J*_@2V_) of 56 A/cm^2^, and effective donor concentration (*N*_d_ − *N*_a_) of 2.3 × 10^14^ cm^−3^ (Figs. 6c, d). Good temperature-dependent performance was also found in the device (Figs. 6e, f). With the increase of temperature, *R*_on_ and *J*_@2V_ became better, demonstrating that the device could work well at high temperature. In their following work, they further deeply investigated the temperature dependence of ideality factor and Schottky barrier height and found that this kind of temperature characteristics can be explained by the Gaussian distribution of barrier height inhomogeneity [61]. In 2018, they further optimized crystal growth parameters and improved the Sn doping concentration (*N*_d_ − *N*_a_ = 2.3 × 10^14^ cm^−3^). The new Pt/*β*–Ga_2_O_3_ SBD device shows markedly improved performance, including forward current density (*J*_@2V_ = 421 A/cm^2^), ON-state resistance (*R*_on_ = 2.9 mΩ cm^2^), a short reverse recovery time (20 ns), and a reverse breakdown voltage higher than 200 V [63]. Their work indicates that EFG grown *β*–Ga_2_O_3_ single crystal is a promising for power device application.

Q. Feng et al. from Xidian University have studied the pulsed laser deposition (PLD) preparation processes and the basic physical properties of the Al-doped *β*–Ga_2_O_3_ film [64–66]. Doping Al is able to tune the bandgap of *β*–Ga_2_O_3_ by incorporating different Al atom ratios. Based on this kind Al-doped film, Ni/*β*-(AlGa)_2_O_3_ SBD device was fabricated and characterized. The Schottky barrier height is 1.33 eV. The current on-off ratio and on-resistance reach 10^11^ and 2.1 mΩ cm^2^, respectively [65]. They also studied the influence of the temperature on the ideality factor and Schottky barrier height and also got the conclusion that these temperature dependence characteristics of *n* and *Φ*_B_ were attributed to the Schottky barrier inhomogeneities by assuming the existence of a Gaussian distribution of the barrier height [66].

**Fig. 7:**
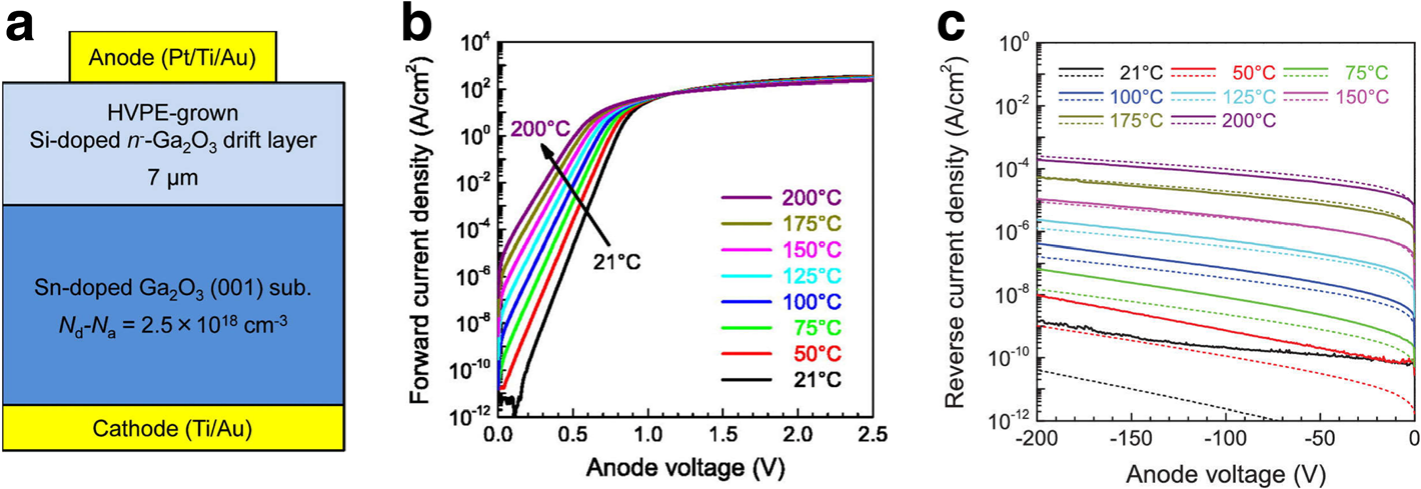
**a** Structure of the SBD device based on the HVPE-grown 7-μm-thick n^−^*-*Ga_2_O_3_ homoepitaxial drift layer on n^+^*-*Ga_2_O_3_ single crystal substrate. **b** Forward J–V characteristics of the device measured at 21–200 °C. **c** Reverse J–V at 21–200 °C (solid and dotted lines represent the experimental and simulated results, respectively). Reprinted from ref. [16]

With the development of the film epitaxy technology, halide vapor-phase epitaxy (HVPE) was utilized to grow Ga_2_O_3_ film. Owing to the advantages of rapid speed of the epitaxy and high quality of the film, HVPE-grown Ga_2_O_3_ is very suitable for fabricating the drift layer of the high-voltage SBD. In 2015, M. Higashiwaki et al. in the National Institute of Information and Communications Technology (NICT) grew 7-μm-thick lightly doped (~ 1 × 10^16^ cm^−3^) epitaxial layer on the heavily doped (*N*_d_ − *N*_a_ = 2.5 × 10^18^ cm^−3^) (001) *β-*Ga_2_O_3_ substrate through HVPE method and further fabricated SBD device. The C–V and I–V characteristics of the device at different temperatures were investigated. The change trend of the Schottky barrier height, threshold voltage, C–V and I–V curves with temperature was pointed out. Figure 7 shows the device structure and the forward and reverse J–V–T curves [16]. It was found that at 21–200 °C, the barrier height kept nearly constant. The forward and reverse current agreed well with the thermionic emission (TE) and thermionic field emission (TFE) model, respectively. Their results demonstrated the potential of the application of the Ga_2_O_3_ SBD in next-generation power devices.

**Fig. 8:**
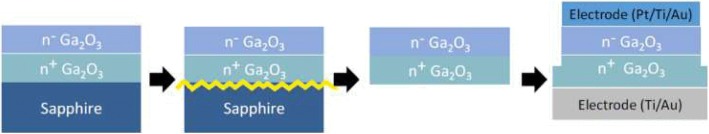
Fabrication processes of the *α*-Ga_2_O_3_ SBD proposed by FLOSFIA Inc. Reprinted from ref. [18, 67]

In 2016, M. Oda et al. in FLOSFIA Inc. published a work about *α*-Ga_2_O_3_ SBD [18]. Through a mist chemical vapor deposition (CVD) technique, i.e., MIST EPITAXY®, they successively grew heavily (3–4 μm thick) and lightly doped *α*-Ga_2_O_3_ films on sapphire (Al_2_O_3_) substrates. After lifting off the *α*-Ga_2_O_3_ layers from the substrates, cathode and anode were deposited on the bottom and top surface of the n^−^*-*Ga_2_O_3_/n^+^*-*Ga_2_O_3_ layers, respectively (Fig. 8). The device with a 2580-nm-thick n^−^*-*Ga_2_O_3_ layer showed a high breakdown voltage of 855 V and an on-resistance of 0.4 mΩ cm^2^. While the device with a thinner (430 nm) n^−^*-*Ga_2_O_3_ layer SBDs exhibited a very low on-resistance of 0.1 mΩ cm^2^ and a breakdown voltage of 531 V. In 2018, they further reported this kind of device conducted with a TO220 package [67]. A junction capacitance of 130 pF was got, so the device showed a better reverse recovery characteristic compared with SiC SBD and Si SBD. At the same time, after package, the device exhibited a thermal resistance of 13.9 °C/W, comparable to that of the SiC SBD with the same package (12.5 °C/W), demonstrating that adopting thin drift layer can effectively compensate the disadvantage of the bad thermal conductivity of Ga_2_O_3_ material. In this report the authors also pointed out that *α*-(Rh,Ga)_2_O_3_ can act as an effective p-type channel layer of *α*-Ga_2_O_3_ devices.

**Fig. 9:**
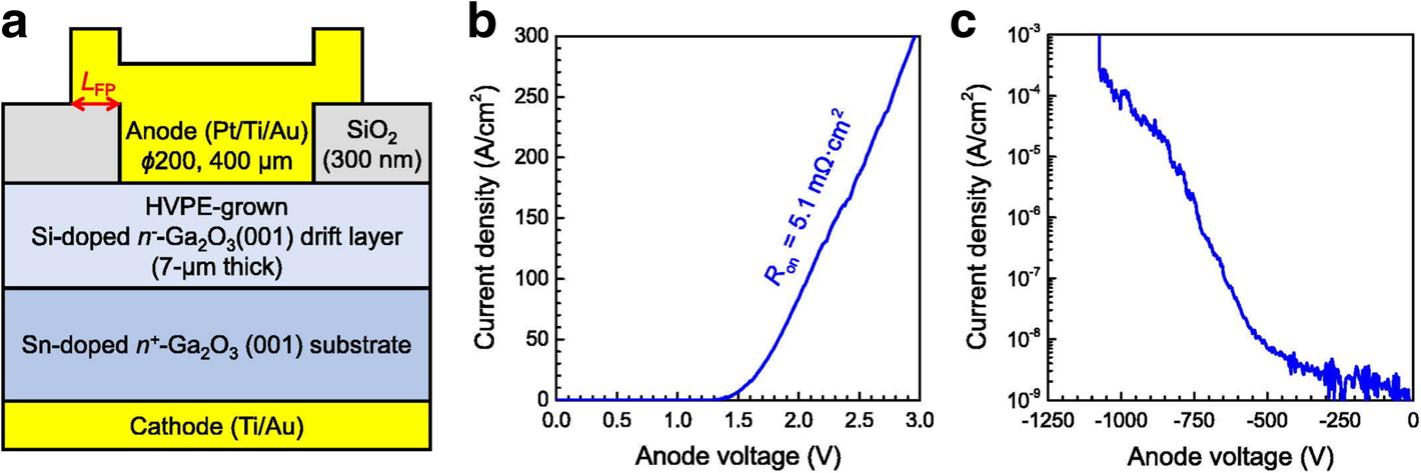
**a** Structure of the SBD with field plate. **b**, **c** Forward and reverse electrical characteristics (*V*_br_ = 1076 V). Reprinted from ref. [68]

In 2017, K. Konishi et al. in NICT reported a Pt/HVPE-n^−^*-*Ga_2_O_3_/(001)n^+^*-*Ga_2_O_3_ SBD device with a breakdown voltage of 1076 V and an on-resistance of 5.1 mΩ cm^2^ (Fig. 9) [68]. Field plate (FP) engineering, a kind of edge termination technology, was first used into Ga_2_O_3_ SBD. By adding an anode connected SiO_2_ FP, the maximum electric field in the entire device structure was kept below the critical field, especially the electric field around the anode can be obviously decreased. Employing this method, both high breakdown voltage and low on-resistance can be achieved. In the same year, a higher breakdown voltage (1600 V) was reported by J. Yang, et al. from the University of Florida in their SBD device with a Ni/MOCVD-n^−^*-*Ga_2_O_3_/(− 201) n^+^*-*Ga_2_O_3_ structure [69], but the on-resistance is very large (25 mΩ cm^2^). No edge termination was used. Their investigation showed that the size of the Schottky electrode had an influence on the breakdown voltage and on-resistance because larger electrode would have more defects and lead to easier breakdown.

**Fig. 10:**
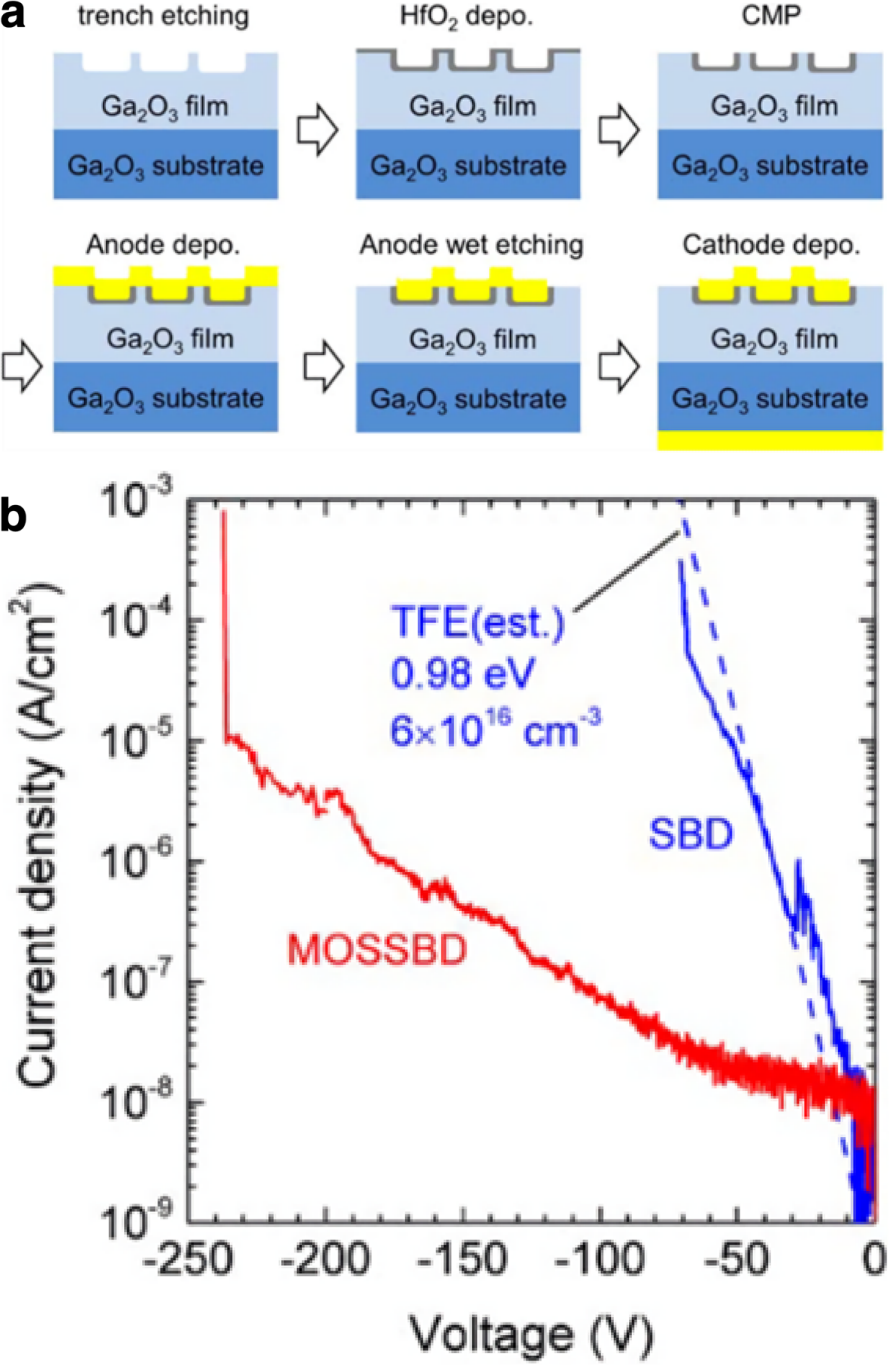
**a** Fabrication processes of the MOS-type Ga_2_O_3_ SBD with trench termination structure. **b** Comparison of the reverse characteristics of the Ga_2_O_3_ SBDs with and without trenches. Reprinted from ref. [70]

In 2017, K. Sasaki et al. from Novel Crystal Technology Inc. first fabricated *β*-Ga_2_O_3_ SBD with trench termination structure (Fig. 10) [70]. By adopting this kind of structure, the electric field in the Schottky junction can be effectively decreased; thus, the leakage current can be greatly reduced while the forward properties are well maintained. The on-resistance of the device was 2.9 mΩ cm^2^, and the breakdown voltage reached about 240 V. At the same time, the threshold voltage was remarkably reduced compared with the previous reports. This work is a valuable exploration on the advanced fabrication process of Ga_2_O_3_ SBD. In the 2nd International Workshop on Gallium Oxide and Related Materials (IWGO 2017) held in Italy, they further reported the improved trench SBD. The threshold voltage decreased to 0.5 V. On-resistance was 2.4 mΩ cm^2^, and breakdown voltage was over 400 V. Compared to the commercialized 600 V SiC SBD, the improved trench Ga_2_O_3_ SBD exhibited superiority in switching loss.

**Fig. 11:**
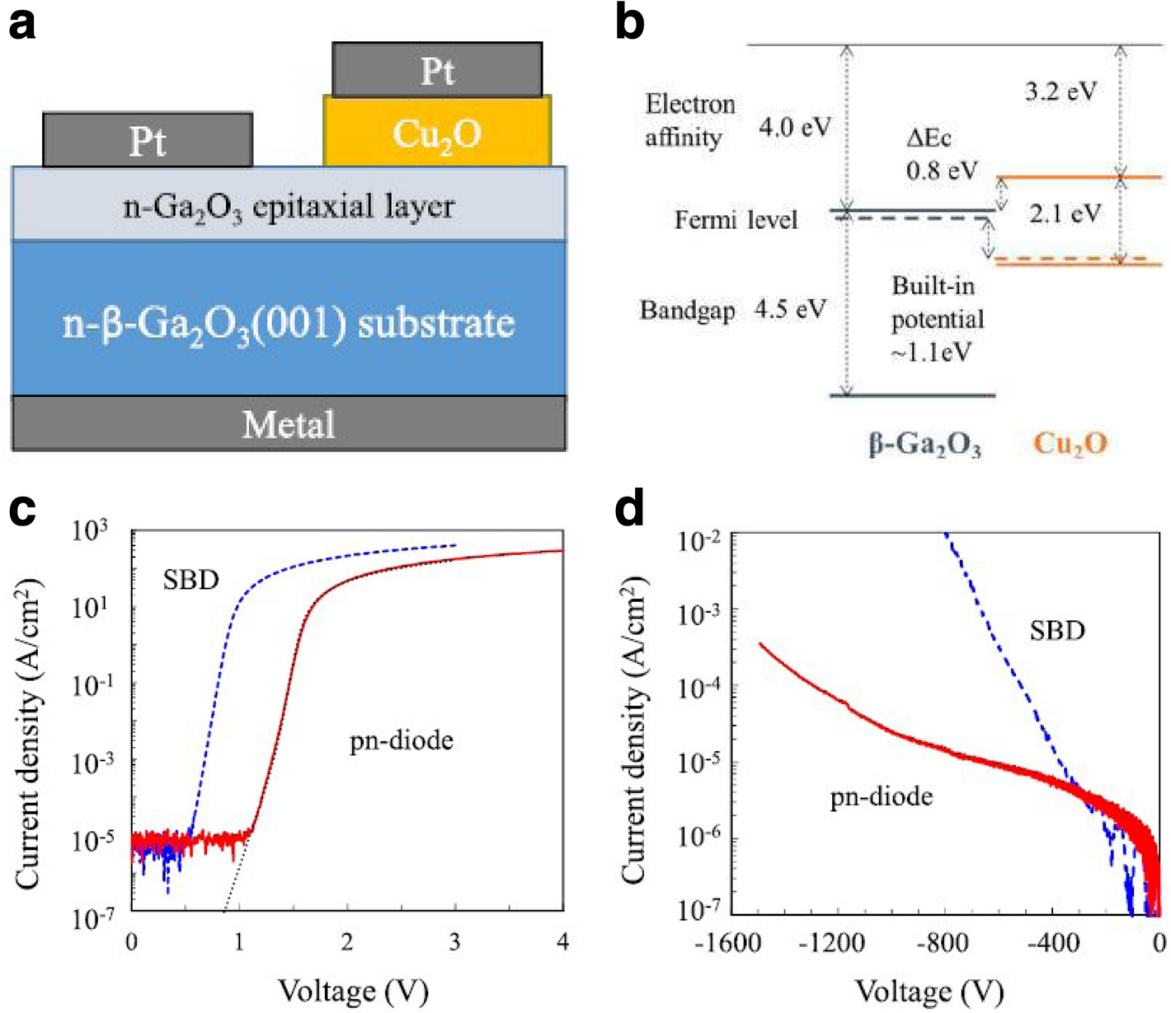
**a** Schematic of the cross-sectional Pt/Ga_2_O_3_ SBD and p-Cu_2_O/n-Ga_2_O_3_ diode. **b** Band diagram of the p-Cu_2_O/n-Ga_2_O_3_ interface. **c**, **d** Forward and reverse J–V characteristics of the SBD and p-n diode. Reprinted from ref. [71]

To date, there has been no effective p-type doping in Ga_2_O_3_, so bipolar devices are not easy to be realized. In 2017, T. Watahiki et al. from Mitsubishi Electric Corporation reported a heterojunction p-Cu_2_O/n-Ga_2_O_3_ p-n diode without local termination structure [71]. Figure 11 shows the schematic, band diagram and J–V curves of this p-n diode. Pt/Ga_2_O_3_ SBD was simultaneously fabricated and measured for comparison. The breakdown voltage of the p-n diode reached as high as 1.49 kV. The on-resistance was 8.2 mΩ cm^2^, much lower than that of the SBD with a thick drift layer reported by J. Yang et al. [69]. So, it can be found that bipolar Ga_2_O_3_ device has a certain advantage over unipolar device in regard to the low on-resistance. This work provides a possible solution for the design Ga_2_O_3_-based bipolar devices. However, this p-n diode exhibited a high threshold voltage (1.7 V). Moreover, in bipolar device, there is the minority carrier storage effect. With the improvement of SBD device structure, this p-n diode appears to show significant competitivity in the aspect of 600–1200 V voltage-resistant level and high frequency. It is believed that with the continuous exploration on the materials, SBD might still be a more effective approach for development of the high-voltage Ga_2_O_3_ device before the successful preparation of p-type Ga_2_O_3_.

**Fig. 12:**
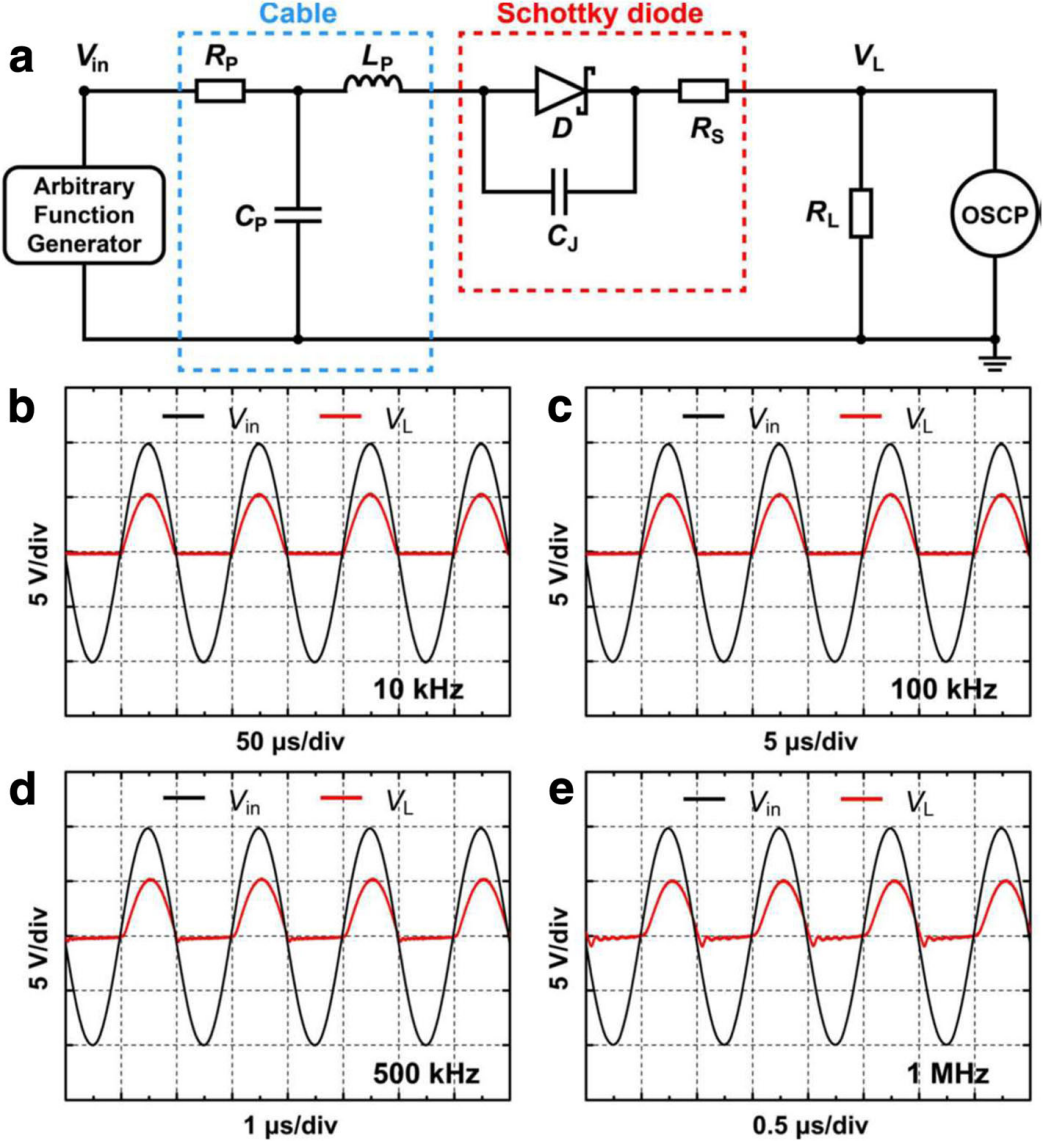
**a** Rectification circuit. **b**–**e** Rectifying effect of Pt/Ga_2_O_3_ SBD on the AC signals under frequency of 10 kHz, 100 kHz, 500 kHz, and 1 MHz. Reprinted from ref. [63]

In practical applications, SBD is usually used to rectify the AC or pulse signals as a rectifier in a circuit. It should work at different frequencies. Q. He et al. from IMECAS investigated the rectification characteristics of the Pt/Ga_2_O_3_ SBD under the AC frequency under 10 kHz to 1 MHz by using a half-wave rectification circuit (Fig. 12) [63]. The testing result proves that the device has the ideal working frequency of 100 kHz, which is equivalent to that of SiC. This work is beneficial for people to further explore how Ga_2_O_3_ Schottky rectifier can operate at higher frequency and also to construct power circuit modules based on Ga_2_O_3_ SBD single device.

Table 2 lists and compares the basic performance parameters of some typical Ga_2_O_3_ Schottky barrier diode reported since 2012. From this table, it is apparent that with the improvement of device structure and fabrication processes, the performances are getting better and better.

**Table 2:** Basic performance parameters of reported Ga_2_O_3_ Schottky barrier diode since 2012

Device structure	*n*	*N*_*d*_ − *N*_a_	*J*_@2 *V*_	*R*_s_	*R*_on_	*qV*_bi_	*q*Φ_b_	*J*_s_	*V*_br_	Structure	Reference
		(cm^−3^)	(A/cm^2^)	(mΩ·cm^2^)	(mΩ·cm^2^)	(eV)	(eV)	(A/cm^2^)	(V)		
Pt/(100)*β*-Ga_2_O_3_/Ti	1.1	2 × 10^17^	421	2.8	2.9	0.63	0.9	2 × 10^−16^	~ 200	Wafer	Our work, 2018 IEEE EDL [63]
Pt/(010)*β*-Ga _2_O_3_/Ti	1.1	2.3 × 10^14^	56	9	12.5	1.07	1.3–1.4	2 × 10^−16^	> 40	Wafer	Our work, 2017 APL [60]
Ni/(100)*β*-(AlGa)_2_O_3_/Ti	2.3	4.5 × 10^18^ (sub)	7.7 (1.7 V)	30.1	63.6	–	0.81	–	–	Epi-layer	Xidian University, China 2018 APL [66]
Ni/(001)*β*-Ga_2_O_3_/Ti	1.03	3.6 × 10^18^ (sub)	–	–	80	–	1.07	–	97	Epi-layer	UF, USA 2018 IEEE Trans. Electron Devices [72]
Mo/(001)*β*-Ga_2_O_3_/Ti	–	5 6 × 10^16^ (5 μm) 6 × 10^18^ (570 μm)	~ 200 (1 V)	–	1.9–2.4	~ 0.5	–	–	> 400	TMBS	Novel Crystal Tecnology, Inc., Japan IWGO 2017
Ni/(−201)*β*-Ga_2_O_3_/Ti	1.07	4 × 10^15^ (10 μm) 3.6 × 10^18^ (650 μm)	–	–	1.6–25	–	1.22	–	~ 1600	Epi-layer	UF, USA 2017 EDL [69]
Cu/(001)*β*-Ga_2_O_3_/Ti	1.1	6 × 10^16^ (7 μm) 2.5 × 10^18^ (350 μm)	~ 210 (1.5 V)	–	2.9	0.7–0.8	1.07	–	230	TMBS	Novel Crystal Tecnology, Inc., Japan 2017 EDL [70]
Ni/(001)*β*-Ga_2_O_3_/Ti	1.08	2 × 10^16^ (10 μm) 3.6 × 10^18^ (650 μm)	–	–	6	–	1.1	–	1016	Epi-layer	UF, USA 2017 APL [73]
Pt/(001)*β*-Ga2O3/Ti	1.03 ± 0.02	1.8 × 10^16^	~ 80	–	5.1	1.32	1.46	–	1076	Field plate	NICT, Japan 2017 APL [68]
Ni/(010)*β*-Ga_2_O_3_/Sn	1.21–3.38	UID (2 μm) 4.1 × 10^18^ (650 μm)	–	–	–	–	0.95–1.01	–	210	Epi-layer	Korea, 2017 SST [74]
Pt/*α*-Ga_2_O_3_/Ti	–	–	3000	–	0.1	1.5–1.6	–	–	531	Film	FLOSFIA, Inc., Japan 2016 APE [18]
	–	–	1350	–	0.4	1.5–1.6	–	–	855	Film	
Ni/(−201)*β*-Ga_2_O_3_/Ti	1.19	2.6 × 10^16^	< 1	51.8	–	–	1.04–1.12	–	–	–	USA, 2016 SST [75]
Pt/(001)*β*-Ga_2_O_3_/Ti	1.03 ± 0.01	1 × 10^16^	> 100	–	3	1.0–1.1	1.12 ± 0.03	–	500	Epi-layer	NICT, Japan 2016 APL [16]
Pt/(010)*β*-Ga_2_O_3_/Ti	1.04–1.06	3 × 10^16^	~ 100	–	7.85	1.23	1.3–1.5	6.5 × 10^−19^	150	Wafer	Japan, 2013 EDL [62]
	–	5 × 10^16^	> 100	–	4.3	1.23	–	9 × 10^−19^	120	Wafer	
Cu/(−201)*β*-Ga_2_O_3_/Ti	1.2–1.4	8 × 10^17^	~ 10	–	–	1.44	0.88–0.95	–	–	Wafer	Germany, 2013 PSS [76]
Au/(100)*β*-Ga_2_O_3_	1.02–1.09	6 × 10^16−^ 8 × 10^17^	< 1	–	–	–	1.07 ± 0.05	–	–	Wafer	Germany, 2012 APL [77]

## Conclusions

Currently, Ga_2_O_3_ SBD is still in its early stage. With the continuous development of fabrication processes, device structure becomes more and more complicated. At the same time, the improvement of the quality of single-crystal substrates and epitaxial films also significantly push forward device performances. However, to date, the development process of Ga_2_O_3_ SBD is very similar to those of previous Si SBD and SiC SBD. Furthermore, the research works on the intrinsic properties of Ga_2_O_3_ materials are still very few. But it is believed that on the basis of its ultrawide bandgap of 4.7–4.9 eV and the development of device structure, Ga_2_O_3_ will better display its unique application value, which requires the joint efforts of the researchers.

## Abbreviations

AC: Alternating current
BFOM: Baliga’s figure of merit
CVD: Chemical vapor deposition
EFG: Edge-defined film-fed growth
FFT: Fast Fourier transform
FP: Field plat
FZ: Floating zone
HRTEM: High-resolution transmission electron microscopy
HVPE: Halide vapor-phase epitaxy
IMECAS: Institute of Microelectronics of Chinese Academy of Sciences
MOCVD: Metal-organic chemical vapor deposition
MOSFET: Metal-oxide-semiconductor field-effect transistor
NICT: National Institute of Information and Communications Technology
PLD: Pulsed laser deposition
SBD: Schottky barrier diode
TE: Thermionic emission
TFE: Thermionic field emission
WBG: Wide bandgap
XRD: X-ray diffraction
